# Author Correction: Structural insights into ligand recognition and activation of the medium-chain fatty acid-sensing receptor GPR84

**DOI:** 10.1038/s41467-023-39757-y

**Published:** 2023-07-05

**Authors:** Heng Liu, Qing Zhang, Xinheng He, Mengting Jiang, Siwei Wang, Xiaoci Yan, Xi Cheng, Yang Liu, Fa-Jun Nan, H. Eric Xu, Xin Xie, Wanchao Yin

**Affiliations:** 1 State Key Laboratory of Drug Research, Shanghai Institute of Materia Medica, Chinese Academy of Sciences, Shanghai, China; 2 School of Pharmaceutical Science and Technology, Hangzhou Institute for Advanced Study, University of Chinese Academy of Sciences, 310024 Hangzhou, China; 3 Shandong Laboratory of Yantai Drug Discovery, Bohai Rim Advanced Research Institute for Drug Discovery, 264117 Yantai, Shandong China; 4 National Center for Drug Screening, Shanghai Institute of Materia Medica, Chinese Academy of Sciences, 201203 Shanghai, China; 5 University of Chinese Academy of Sciences, 100049 Beijing, China; 6 School of Chinese Materia Medica, Nanjing University of Chinese Medicine, 210023 Nanjing, China; 7 School of Life Science and Technology, ShanghaiTech University, 201210 Shanghai, China; 8 Zhongshan Institute for Drug Discovery, Shanghai Institute of Materia Medica, Chinese Academy of Sciences, 528400 Guangdong, China

**Keywords:** Cryoelectron microscopy, Inflammatory diseases, G protein-coupled receptors

Correction to: *Nature Communications* 10.1038/s41467-023-38985-6, published online 06 June 2023

The original version of this Article contained an error in Ref. 19, which was incorrectly given as: Pillaiyar, T. et al. 6-(Ar)alkylamino-substituted uracil derivatives: lipid mimetics with potent activity at the orphan G protein-coupled receptor 84 (GPR84). *ACS Omega* **3**, 3365–3383 (2018). The correct Ref. 19 is: Liu, Y., et al. Design and Synthesis of 2-Alkylpyrimidine-4,6-diol and 6-Alkylpyridine-2,4-diol as potent GPR84 agonists. *ACS Med. Chem. Lett.* **7**, 579–583 (2016). This has been corrected in the PDF and HTML versions of the Article.

