# Supplementary material for: Structural insights into ligand recognition and activation of the medium-chain fatty acid-sensing receptor GPR84
Source: Nat Commun. 2023 Jun 6;14:3271. doi: 10.1038/s41467-023-38985-6 (PMC10241960; doi:10.1038/s41467-023-38985-6)
Supplement: Supplementary file 1 — Supplementary Information [file 41467_2023_38985_MOESM1_ESM.pdf]

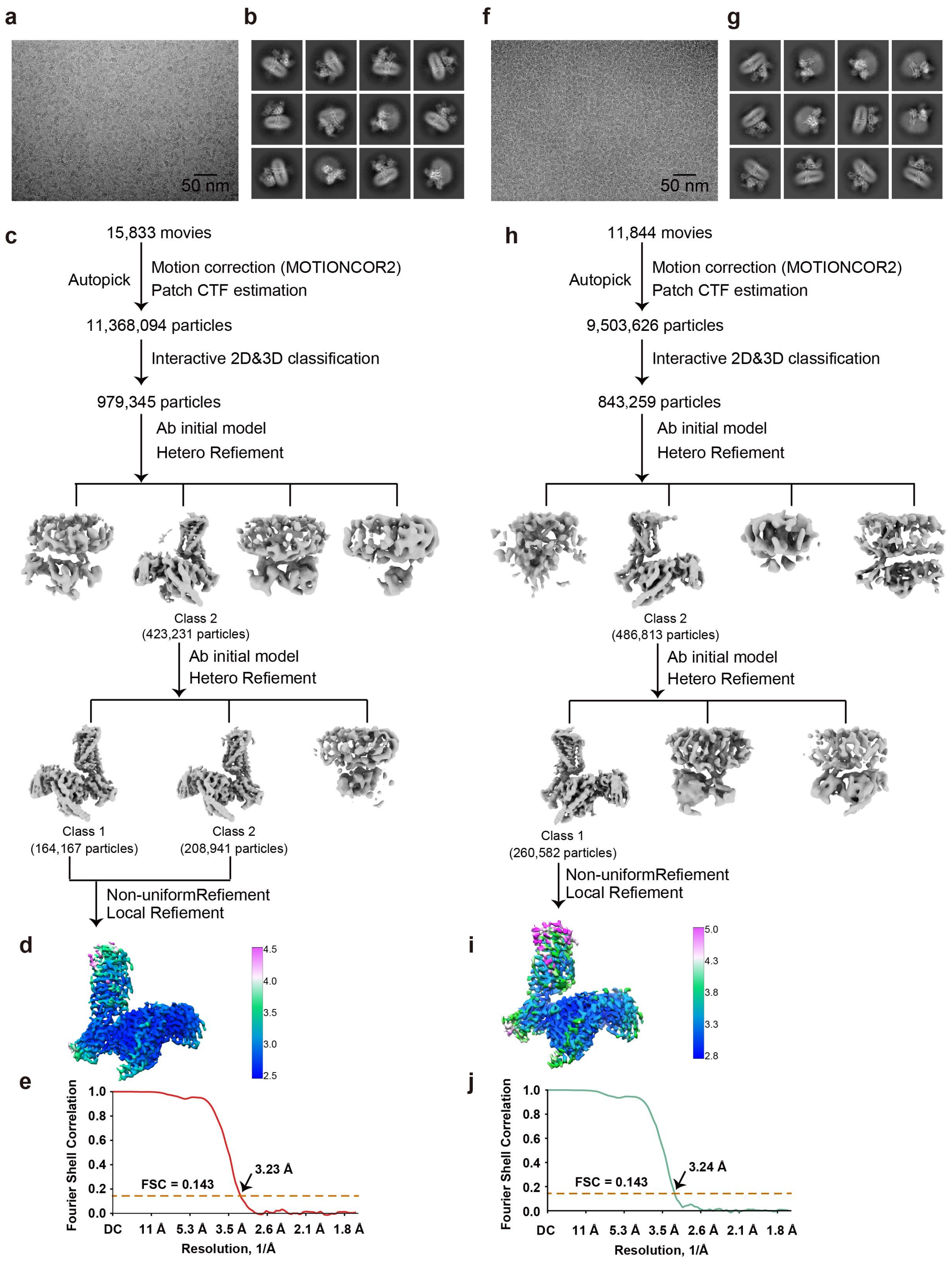

Supplementary Fig. 1 Cryo-EM images and single-particle reconstruction of the LY237-GPR84-Gα_i complex and GPR84-Gα_i complex with no ligand modeled. **a-c,** Cryo-EM micrograph, reference-free 2D class averages, and flowchart of cryo-EM data analysis of the LY237-GPR84 complex. **d,** Cryo-EM map of the LY237-GPR84 complex, colored by local resolutions from 2.5 Å (blue) to 4.5 Å

(purple). **e**, "Gold-standard" Fourier shell correlation (FSC) curve, indicates that the overall resolution of the electron density map of the LY237-GPR84-Gα_i complex is 3.23 Å. **f-h,** Cryo-EM micrograph, reference-free 2D class averages, and flowchart of cryo-EM data analysis of the GPR84-Gαi complex with no ligand modeled. **i,** Local resolutions map of the GPR84-Gαi complex with no ligand modeled, colored by resolution from 2.8 Å (blue) to 5.0 Å (purple). **j**, "Gold-standard" Fourier shell correlation (FSC) curve indicates that the overall resolution of the electron density map of the GPR84-Gαi complex with no ligand modeled complex is 3.24 Å.

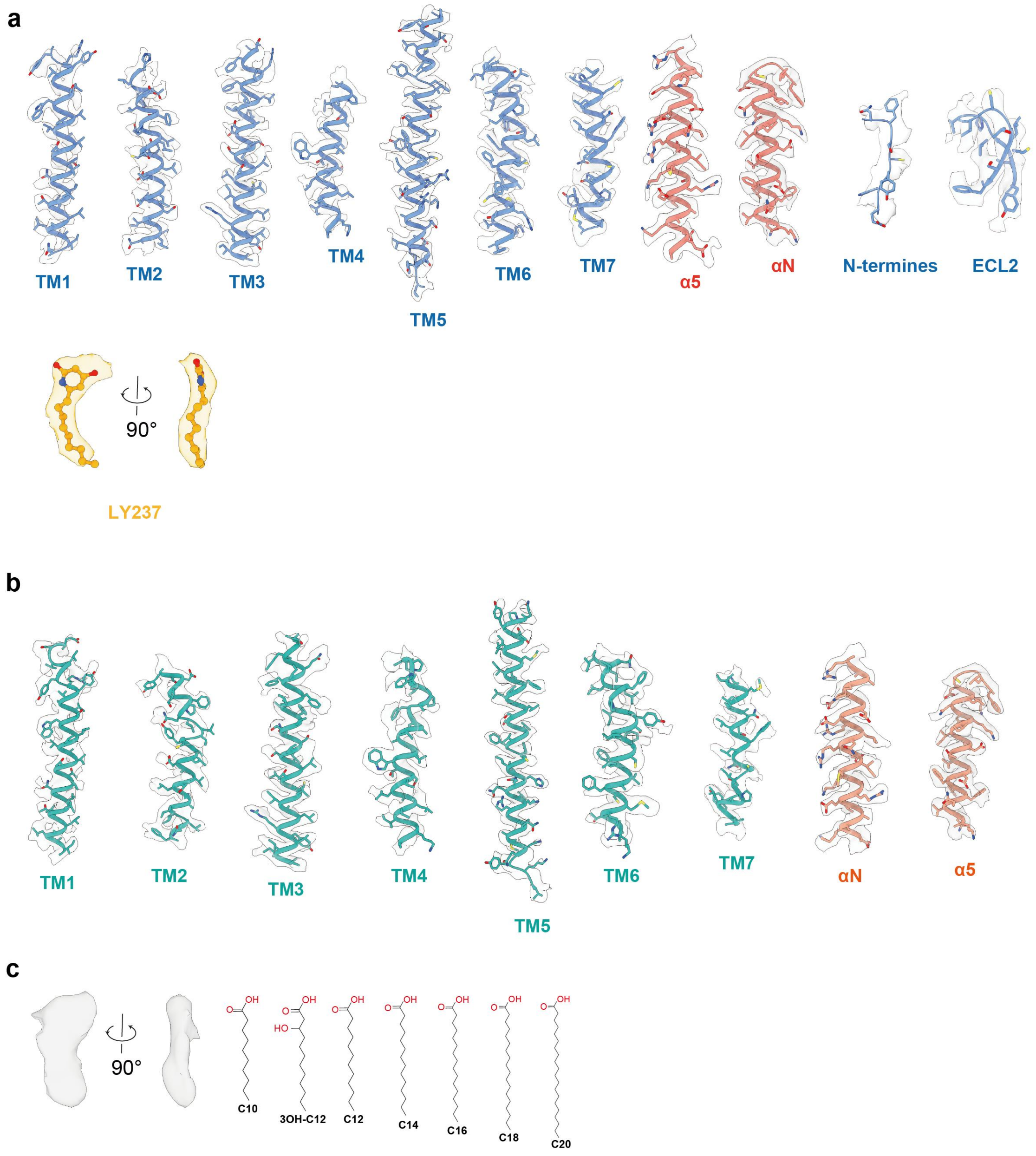

Supplementary Fig. 2 Cryo-EM image density maps

a, LY237 activated GPR84 TMD density maps in the LY237-GPR84-$G\alpha_i$ complex; **b**, GPR84 TMD density maps in the GPR84-Gαi complex with no ligand modeled complex; **c**, Additional density in the binding pocket of GPR84-Gαi complex with no ligand modeled complex, and diagram of free fatty acid with different carbon length.

a

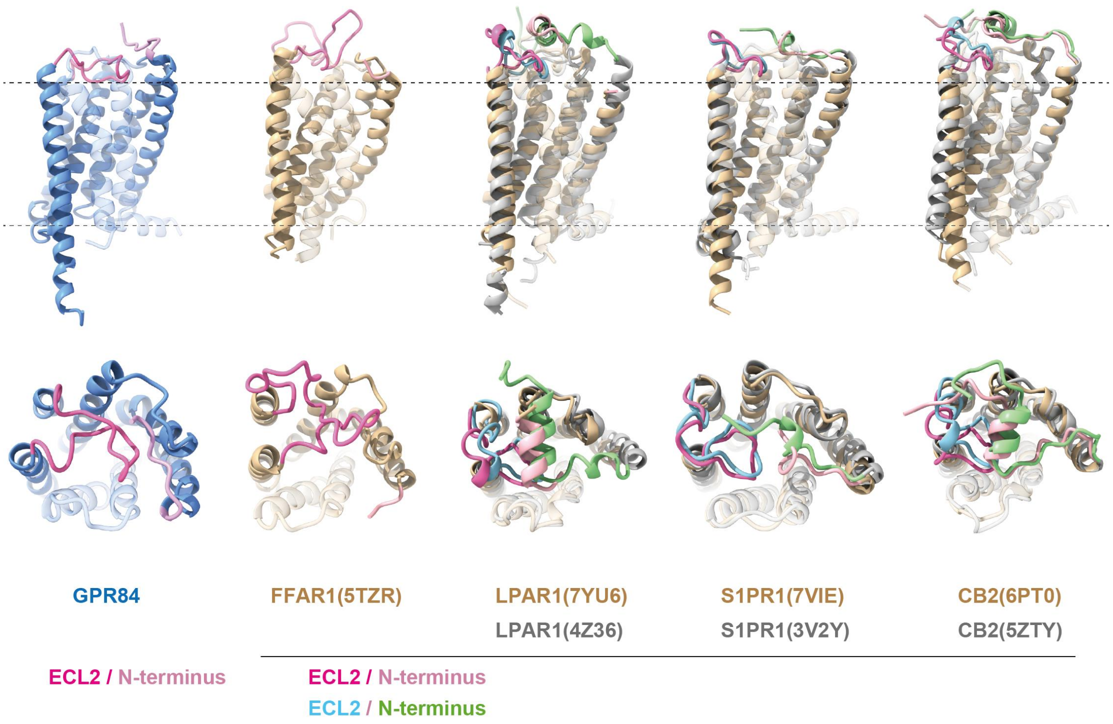

b

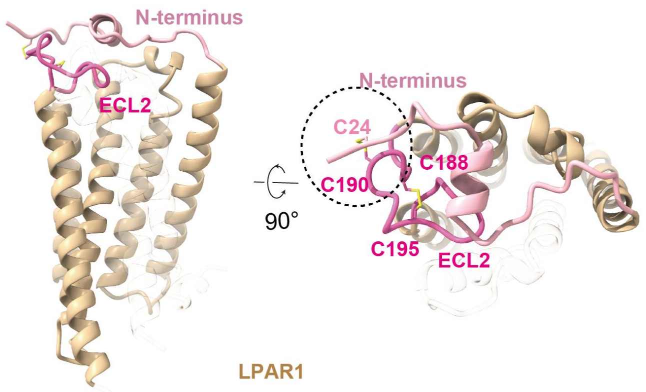

Supplementary Fig. 3. Structural comparison of GPR84 with other receptors

a, Downward movement conformation of ECL2 of GPR84 compared with FFAR1(PDB code: 5TZR), LPAR1(PDB code: 7YU6), S1PR1(PDB code: 7VIE), CB2(PDB code: 6PT0); **b**, Side view (left panel) and top view (right panel) of LPA_1 in activate state. The ECL2 (hot pink), N-terminus (light pink), and disulfide bridge (shown in stick) are highlighted.

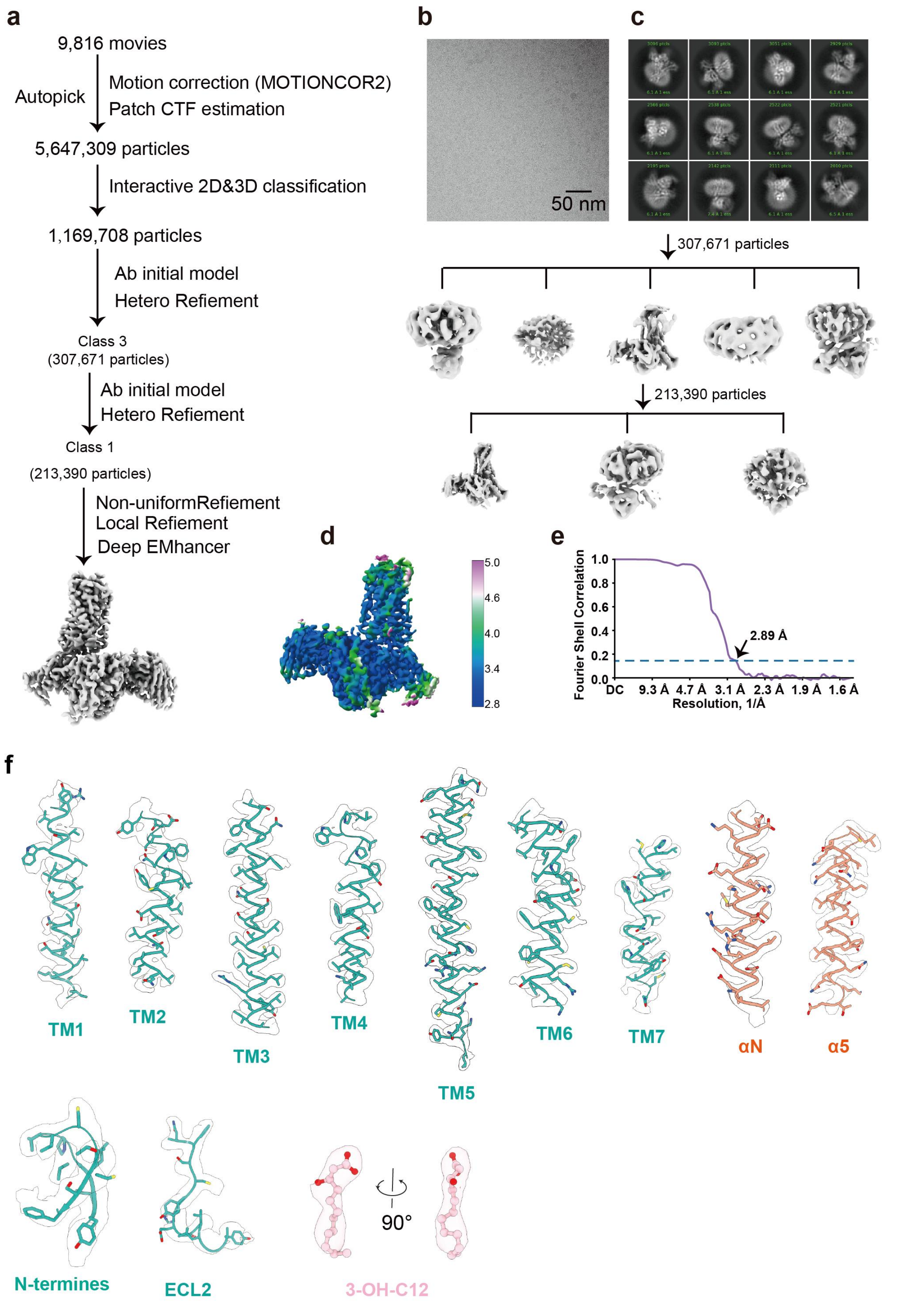

Supplementary Fig. 4 Cryo-EM images and single-particle reconstruction of 3-OH-C12 activated GPR84-Gα_i complex

a, Flowchart of cryo-EM data analysis of the 3-OH-C12-GPR84 complex. **b-c,**

Representative cryo-EM micrograph, reference-free 2D class averages, and 3D classifications of the 3-OH-C12-GPR84 complex. **d,** Cryo-EM map of the 3-OH-C12-GPR84 complex, colored by local resolutions from 2.8 Å (blue) to 5.0 Å (purple). **e**, “Gold-standard” Fourier shell correlation (FSC) curve, indicates that the overall resolution of the electron density map of the3-OH-C12-GPR84-Gα_i complex is 2.89 Å. **f,** 3-OH-C12 activated GPR84 TMD density maps in the 3-OH-C12-GPR84-Gα_i complex.

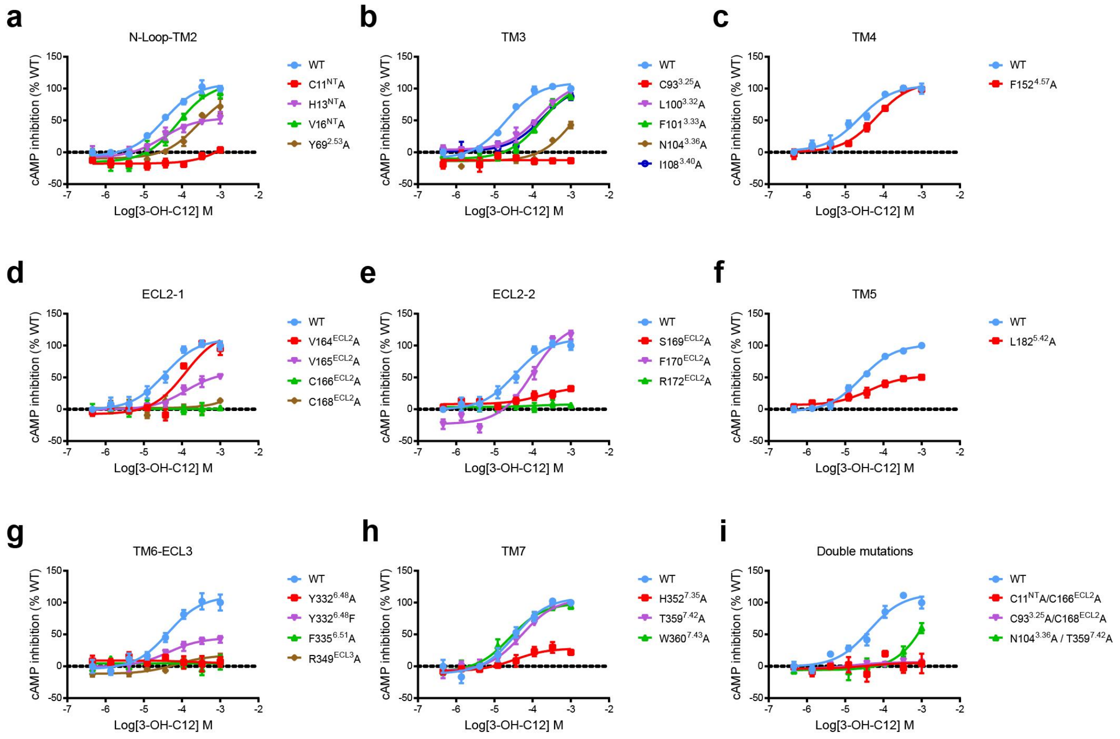

Supplementary Fig. 5. Mutagenesis data of 3-OH-C12 induced GPR84 activation.

a-i, Dose-response curves of 3-OH-C12 in activating GPR84 with various mutations with cAMP assay. Data are presented as means ± SEM with a minimum of three technical replicates, which performed in triplicates. The data were normalized according to the maximal response of 3-OH-C12 induced WT-GPR84 activation. Source data are provided as a Source Data file.

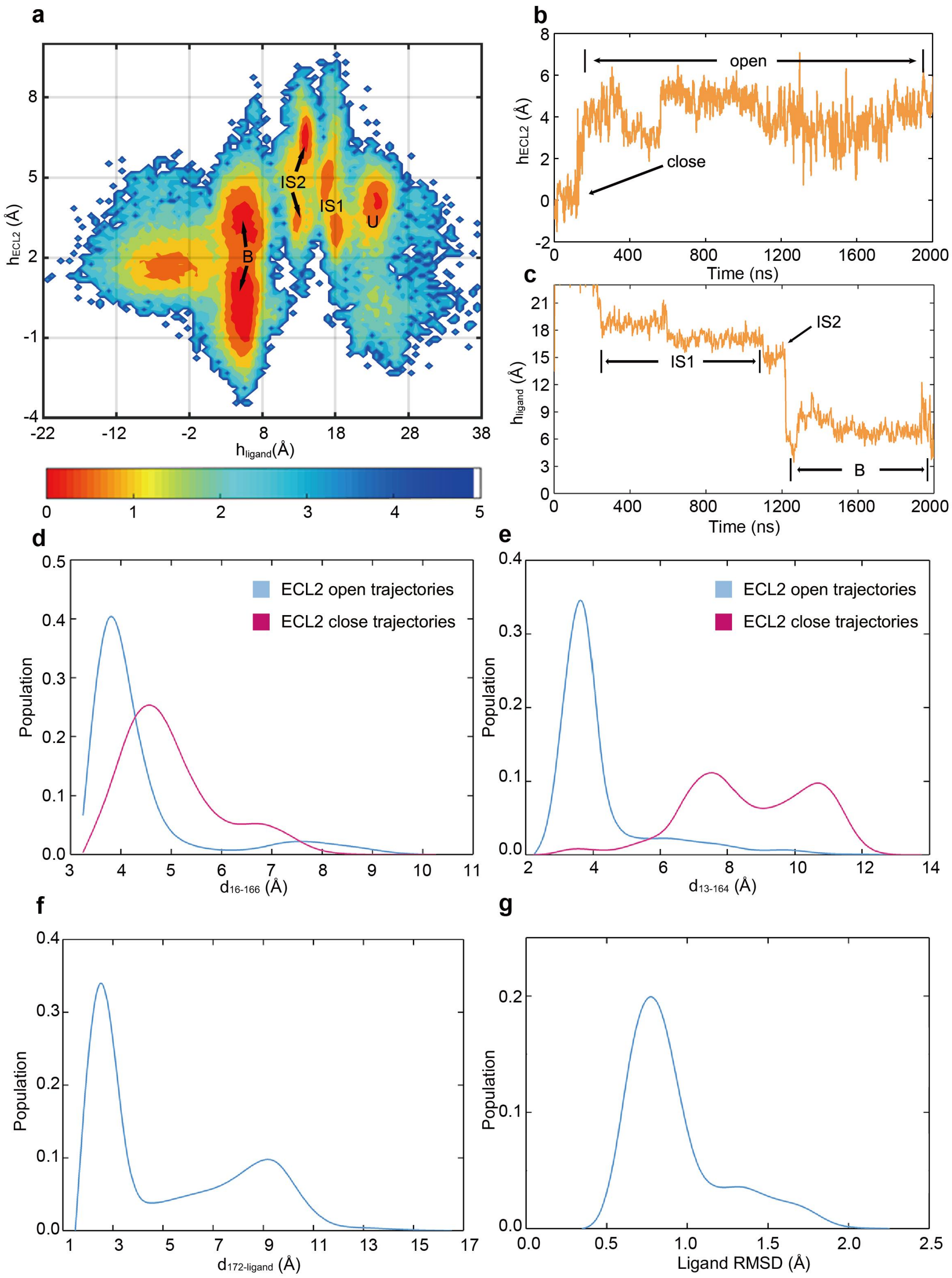

Supplementary Fig. 6. The opening of ECL2 and the entry process of LY237.

a, The free energy landscape of ECL2 open and ligand entry process in LiGaMD simulations. X axis is the difference of Z coordinate between the ligand and the Cα of Y332$^{6.48}$ (h_{ligand}), while Y axis is the difference of Z coordinate between the Cα of V164^{ECL2} in simulations and cryo-EM structure (h_{ECL2}). Black arrows show the unbounded (U), intermediate state 1 (IS1), intermediate state 2 (IS2) and bounded (B)

states. Red rectangles and arrows point out close and open conformations of ECL2. **b**, Time-course variations of $d_{164\text{-}16}$ in the representative trajectory. The close and open states were identified by arrows. **c,** Time-course variations of h_{ligand} in the representative trajectory. The IS1, IS2, and B states were identified by arrows. **d**, The distribution of the minimal distance between the sidechains of V16 and C166 ($d_{16\text{-}166}$) on ECL2 open trajectories (blue) and ECL2 close trajectories (magenta)**. e**, The distribution of the minimal distance between H13 sidechain N and V164 mainchain O ($d_{13\text{-}164}$) on ECL2 open trajectories (blue) and ECL2 close trajectories (magenta). **f**, The distribution of the minimal distance between R172 sidechain N and O on LY237 on conventional MD simulations after LiGaMD. **g**, The distribution of the root mean square deviation (RMSD) between conventional MD and cryo-EM poses. Source data are provided as a Source Data file.

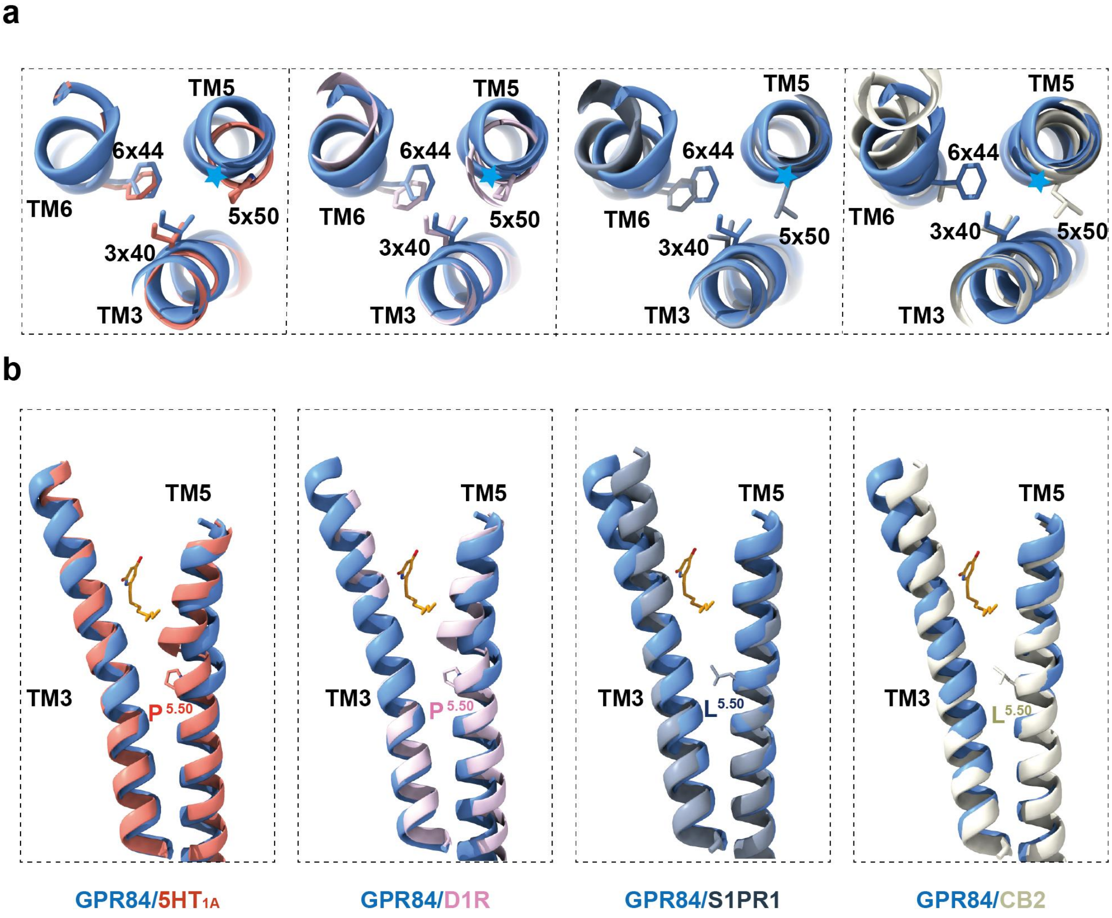

Supplementary Fig. 7. Un-conservative characteristics of the PIF motif in GPR84

a, Structural comparison of GPR84 with 5HT1A (PDB code: 7E2Y), D1R (PDB code: 7JVQ), CB2 (PDB code: 6PT0), S1PR1 (PDB code: 7EVY) receptors in the PIF motif. Light blue star is the location of G190 in GPR84. **b**, Structural comparison of the conformation of TM5 in GPR84 with 5HT1A (PDB code: 7E2Y), D1R (PDB code: 7JVQ), CB2 (PDB code: 6PT0), S1PR1 (PDB code: 7EVY) receptors.

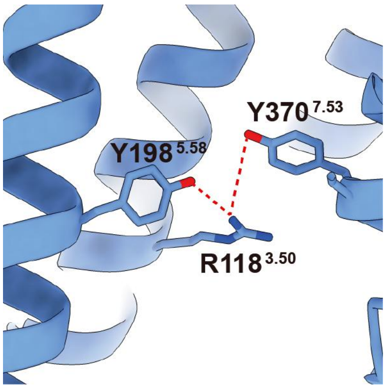

Supplementary Fig. 8. Key interactions of R118$^{3.50}$ in GPR84

Detailed interactions of R118$^{3.50}$ with Y198$^{5.58}$ and Y370$^{7.53}$.

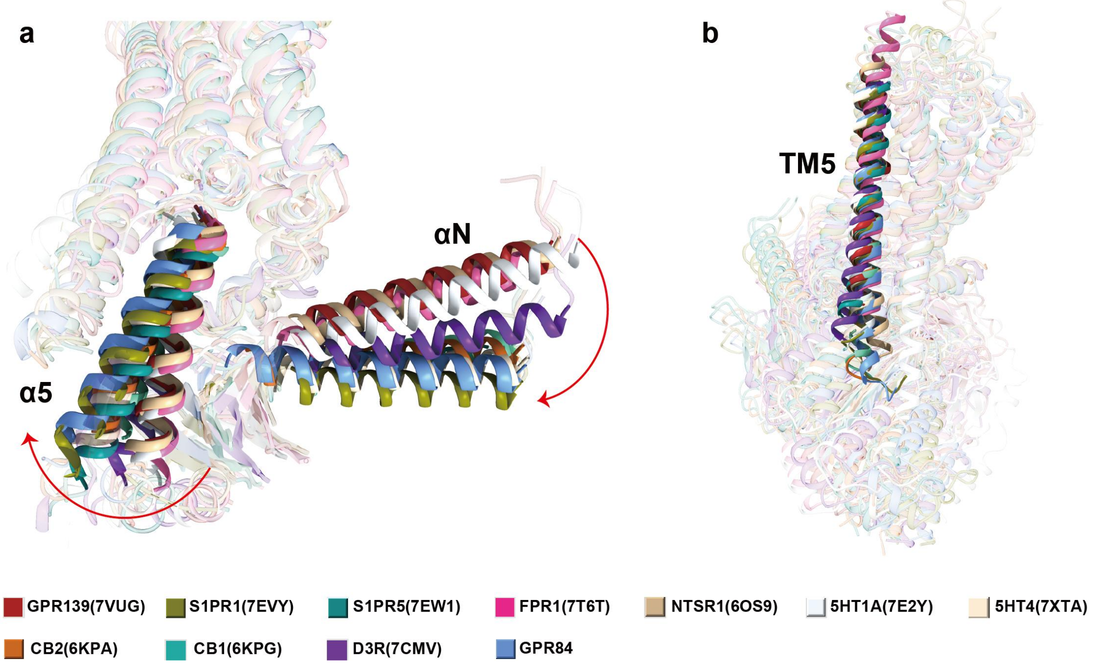

Supplementary Fig. 9. Structural comparison of the coupling mode of GPR84 with other Gα_i coupled receptors.

a, Structural comparison of other Gα_i coupled receptors, focus on the rotate of αN and α5. **b**, Structural comparison shown that the TM5 helix length of GPR84 is longest between compared receptors

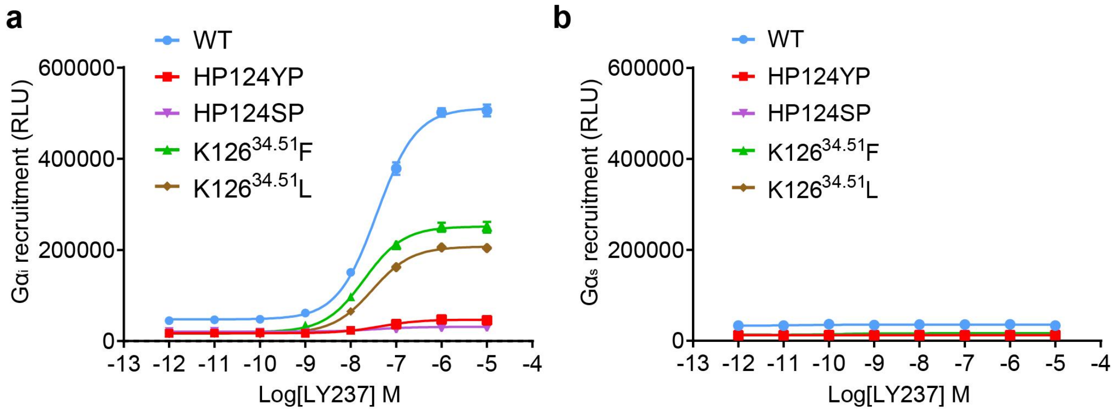

Supplementary Fig. 10. Mutagenesis data of LY237 induced $G\alpha_i$/$G\alpha_s$ coupling of GPR84

a-b, Dose-response curves of LY237 in inducing $G\alpha_i$ (a) and $G\alpha_s$ (b) recruitment to GPR84 in HEK293 cells. Data were presented as means ± SEM with a minimum of three technical replicates, which performed in triplicates. RLU, relative luminescence units. HP124YP: HPKLFPQ from GPR84 replaced by YPLHSRF from galanin receptor 2 (PDB code: 7WQ4); HP124SP: HPKLFPQ from GPR84 replaced by SPFKYQ from beta2 adrenergic receptor (PDB code: 3SN6). Source data are provided as a Source Data file.

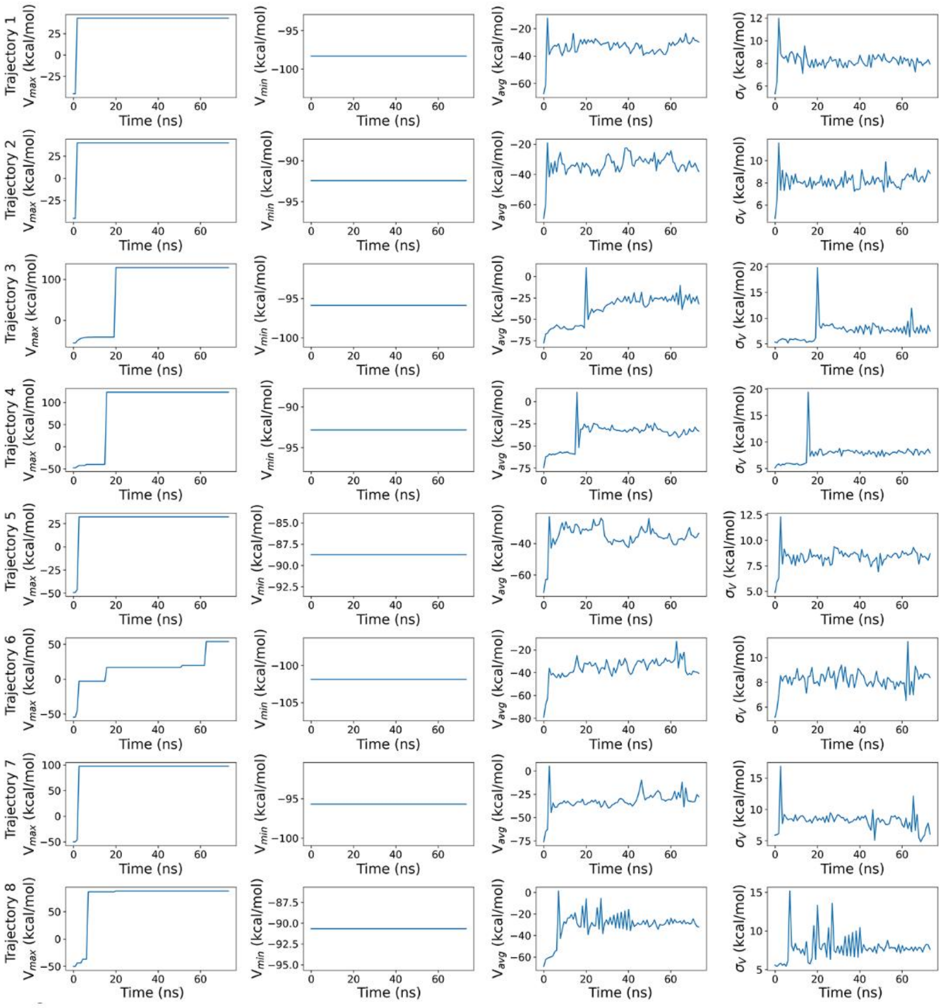

Supplementary Fig. 11. The time-course figure for V_{max}, V_{min}, V_{avg}, and σ_V of ligand binding potential energy during 73.2 ns parameter sampling.

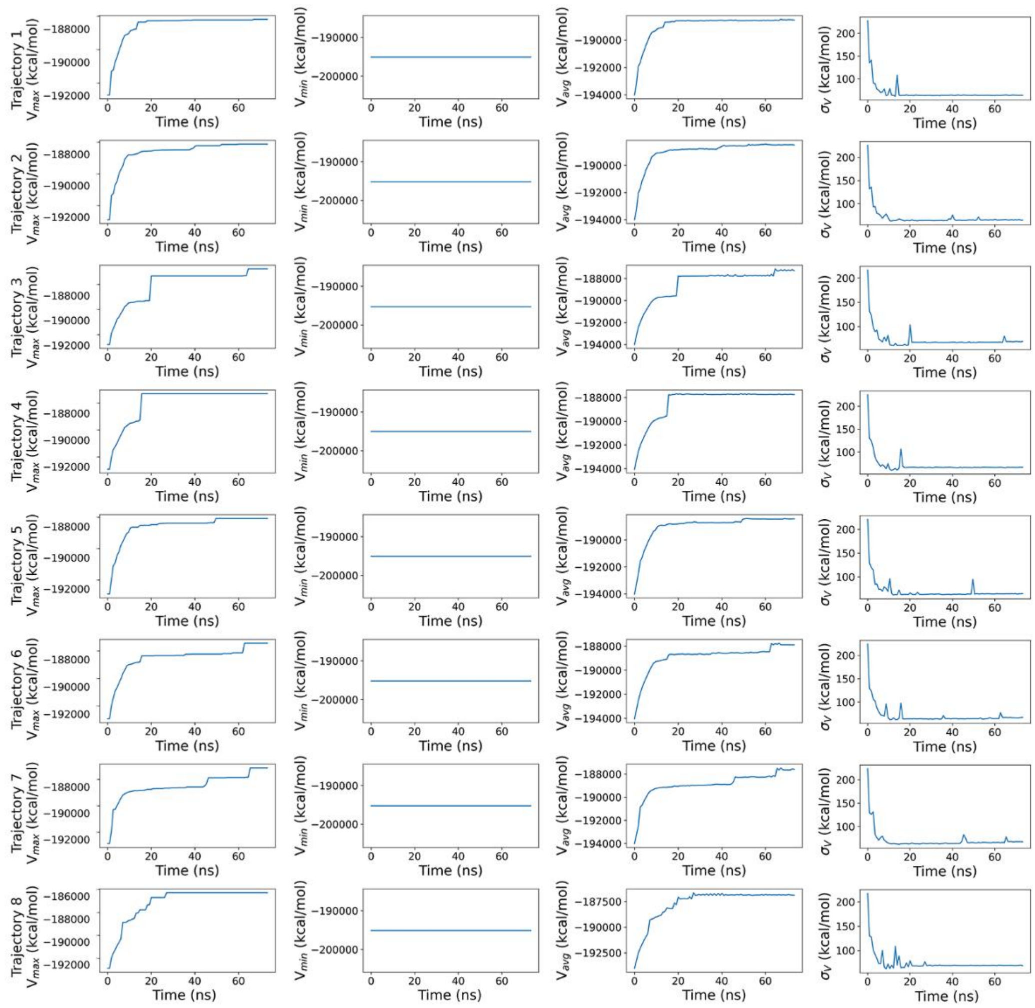

Supplementary Fig. 12. The time-course figure for V_{max}, V_{min}, V_{avg}, and σ_V of remaining potential energy during 73.2 ns parameter sampling.

Supplementary Table 1, Expression levels and ligand-mediated activation of WT and mutant GPR84.

Residue Number	GPR84 mutations	LY237		3-OH-C12		Expression(%WT)
		pEC_{50} ± S.E.M.	E_{max} ± S.E.M.(%WT)	pEC_{50} ± S.E.M.	E_{max} ± S.E.M.(%WT)	
	WT	9.85±0.06	100.00±4.82	4.41±0.08	100.00±3.71	100.00±3.11
N-Loop	C11A	ND	20.39±5.41	ND	3.71±4.23	72.98±1.01
N-Loop	H13A	9.27±0.10	46.58±2.53	4.54±0.19	54.15±9.19	80.06±2.44
N-Loop	V16A	9.25±0.04	86.20±5.00	4.10±0.05	93.62±8.65	85.19±0.11
2.53	Y69A	8.85±0.3	71.4±13.85	3.55±0.12	71.74±12.05	90.64±1.52
3.25	C93A	ND	-13.12±3.36	ND	-13.21±3.07	37.49±3.51
3.32	L100A	8.96±0.06	85.13±2.66	3.90±0.12	96.28±2.40	109.22±0.66
3.33	F101A	8.18±0.22	83.52±5.42	3.83±0.09	87.65±1.83	101.09±4.20
3.36	N104A	8.17±0.10	57.74±3.45	ND	45.17±6.59	95.60±3.99
3.40	I108A	8.67±0.12	85.90±3.54	3.79±0.09	88.47±3.51	78.83±3.68
4.57	F152A	9.29±0.17	85.90±5.80	4.15±0.07	98.91±2.47	85.52±0.30
ECL2	V164A	8.98±0.12	94.33±7.46	3.90±0.03	95.44±10.17	85.17±3.94
ECL2	V165A	8.17±0.26	41.34±5.4	3.98±0.05	51.29±3.17	121.63±1.90
ECL2	C166A	ND	-5.89±1.79	ND	2.37±1.49	70.15±1.90
ECL2	C168A	ND	2.97±7.45	ND	13.54±2.95	38.27±3.76
ECL2	S169A	7.47±0.22	33.27±3.66	3.87±0.11	32.67±1.88	106.52±2.01
ECL2	F170A	9.09±0.19	93.6±9.85	3.93±0.18	116.24±9.63	95.88±3.41
ECL2	R172A	ND	32.83±4.04	ND	6.87±0.66	123.87±3.60
5.42	L182A	8.78±0.15	63.1±5.06	4.40±0.16	50.31±1.55	98.90±1.20
6.48	Y332A	ND	0.87±4.18	ND	6.98±7.79	63.80±2.17
6.48	Y332F	9.39±0.05	33.05±3.09	4.34±0.26	42.20±5.41	74.60±0.57
6.51	F335A	ND	36.30±6.9	ND	5.35±2.61	120.85±1.49
ECL3	R349A	8.16±0.20	25.07±4.64	4.34±0.15	14.55±5.19	77.85±3.00
7.35	H352A	8.27±0.29	36.15±9.15	4.26±0.2	21.96±3.44	60.27±2.01
7.42	T359A	9.84±0.21	95.58±2.25	4.35±0.12	98.71±1.74	73.43±1.31
7.43	W360A	9.74±0.32	26.36±2.54	4.52±0.07	93.70±3.91	86.07±2.95
-	C11A/C166A	ND	8.45±0.56	ND	4.43±15.31	71.78±2.46
-	C93A/C168A	ND	-8.02±0.67	ND	5.21±5.14	36.30±1.28
-	N104A/T359A	7.50±0.17	52.06±14.49	ND	59.88±7.93	75.71±4.04

The wild type (WT)and mutants of GPR84 discussed in this manuscript were individually analyzed. The affinities are derived from at least 3 independent experiments using cAMP assay. The expression level of mutant GPR84 were normalized to wild-type GPR84 as 100 %, respectively. Each data represents mean ± standard error of the mean (S.E.M.).Source data are available online. Definitions: ND - no determination; ND indicates that the activation level is too low to determine EC_{50} values.

Supplementary Table 2, Expression levels and ligand-mediated activation of WT and mutant GPR84.

Residue Number	GPR84 mutations	LY237		Expression(%WT)
		pEC_{50} ± S.E.M.	E_{max} ± S.E.M.(%WT)	
	WT	7.42±0.02	100.00±2.84	100.00±0.51
34.51	K126A	7.55±0.03	86.99±0.85	106.37±1.5
34.51	K126F	7.7±0.01	44.47±2.62	69.48±2.85
34.51	K126L	7.53±0.03	34.56±1.17	76.49±1.76
-	HP124SP	ND	-2.85±0.35	35.69±0.6
-	HP124YP	ND	0.24±0.51	42.50±1.84
5.75	Y215A	7.22±0.18	111.46±9.3	98.79±2.82
-	Delt210-215	7.53±0.02	109.79±2.87	139.9±1.38

The wild type (WT)and mutants of GPR84 discussed in this manuscript were individually analyzed. The affinities are derived from at least 3 independent experiments using $G\alpha_i$ recruitment assay. The expression level of mutant GPR84 were normalized to wild-type GPR84 as 100 %, respectively. Each data represents mean ± standard error of the mean (S.E.M.).Source data are available online. Definitions: ND - no determination; ND indicates that the activation level is too low to determine EC_{50} values.
HP124YP: HPKLFPQ from GPR84 replaced by YPLHSRF from galanin receptor 2 (PDB code: 7WQ4); HP124SP: HPKLFPQ from GPR84 replaced by SPFKYQ from beta2 adrenergic receptor (PDB code: 3SN6).

Supplementary Table 3, Cryo-EM data collection, refinement and validation statistics.

	LY237-GPR84-Gαi complex	GPR84-Gαi complex with no ligand modeled	3-OH-C12-GPR84-Gαi complex
Magnification	105,000	105,000	96,000
Voltage (kV)	300	300	300
Electron exposure (e–/Å^2)	50	50	50
Defocus range (μm)	-1.0 to -2.0	-1.0 to -2.0	-1.0 to -2.0
Pixel size (Å)	0.824	0.824	0.73
Symmetry imposed	C1	C1	C1
Initial particle images (no.)	11,368,094	9,503,626	5,647,309
Final particle images (no.)	373,108	260,582	213,390
Map resolution (Å)	3.23	3.24	2.89
FSC threshold	0.143	0.143	0.143
Map resolution range (Å)	2.8-5.0	2.8-5.0	2.8-5.0
**Refinement**			
Initial model used (PDB code)	AlphaFold GPR84	AlphaFold GPR84	AlphaFold GPR84
Map sharpening *B* factor (Å^2)	-107.9	-122.9	-117
Model composition			
Non-hydrogen atoms	8,822	8,849	8,864
Protein residues	1,131	1,137	1,137
Ligands	1		1
B factors (Å^2)			
Protein	46.04	64.92	124.50
Ligand	20.00		97.13
R.m.s. deviations			
Bond lengths (Å)	0.004	0.005	0.005
Bond angles (°)	0.951	0.976	1.013(0)
Validation			
MolProbity score	1.28	1.51	1.60
Clashscore	4.95	4.76	5.84
Poor rotamers (%)	0.00	0.00	0.00
Ramachandran plot			
Favored (%)	97.93	96.07	95.98
Allowed (%)	2.07	3.93	4.02
Disallowed (%)	0.00	0.00	0.00
