# Supplementary material for: Structural insights into ligand recognition and activation of the medium-chain fatty acid-sensing receptor GPR84
Source: Nat Commun. 2023 Jun 6;14:3271. doi: 10.1038/s41467-023-38985-6 (PMC10241960; doi:10.1038/s41467-023-38985-6)
Supplement: Supplementary file 3 — Description of Additional Supplementary Files [file 41467_2023_38985_MOESM3_ESM.pdf]

File name: Supplementary Data 1

Description: Results of MS analysis on GPR84-Gαi complex with no ligand added in the positive and negative ion modes.

File name: Supplementary Movie 1

Description: The process of ligand entry during LiGaMD and the stable pose of the ligand in the following conventional MD The upper left portion of the video shows the state during the entry process, while the upper right indicates the type of simulation being performed. Key residues involved in the ligand entry and stable pose are displayed as sticks with labels, and ECL2 and extracellular TM6-7 are represented as green and purple cartoons, respectively.
