# Supplementary material for: Structural insights into ligand recognition and activation of the medium-chain fatty acid-sensing receptor GPR84
Source: Nat Commun. 2023 Jun 6;14:3271. doi: 10.1038/s41467-023-38985-6 (PMC10241960; doi:10.1038/s41467-023-38985-6)
Supplement: Supplementary file 6 — Reporting Summary [file 41467_2023_38985_MOESM6_ESM.pdf]

nature portfolio

Corresponding author(s): Wanchao Yin
Xin Xie
H. Eric Xu

Last updated by author(s): May 11, 2023

Reporting Summary

Nature Portfolio wishes to improve the reproducibility of the work that we publish. This form provides structure for consistency and transparency in reporting. For further information on Nature Portfolio policies, see our Editorial Policies and the Editorial Policy Checklist.

Statistics

For all statistical analyses, confirm that the following items are present in the figure legend, table legend, main text, or Methods section.

n/a	Confirmed	
☐	☒	The exact sample size (*n*) for each experimental group/condition, given as a discrete number and unit of measurement
☐	☒	A statement on whether measurements were taken from distinct samples or whether the same sample was measured repeatedly
☐	☒	The statistical test(s) used AND whether they are one- or two-sided<br>*Only common tests should be described solely by name; describe more complex techniques in the Methods section.*
☒	☐	A description of all covariates tested
☒	☐	A description of any assumptions or corrections, such as tests of normality and adjustment for multiple comparisons
☐	☒	A full description of the statistical parameters including central tendency (e.g. means) or other basic estimates (e.g. regression coefficient) AND variation (e.g. standard deviation) or associated estimates of uncertainty (e.g. confidence intervals)
☒	☐	For null hypothesis testing, the test statistic (e.g. *F*, *t*, *r*) with confidence intervals, effect sizes, degrees of freedom and *P* value noted<br>*Give P values as exact values whenever suitable.*
☒	☐	For Bayesian analysis, information on the choice of priors and Markov chain Monte Carlo settings
☒	☐	For hierarchical and complex designs, identification of the appropriate level for tests and full reporting of outcomes
☒	☐	Estimates of effect sizes (e.g. Cohen's *d*, Pearson's *r*), indicating how they were calculated

Our web collection on statistics for biologists contains articles on many of the points above.

Software and code

Policy information about availability of computer code

Data collection	Cryo-EM data collection on the Titan Krios using EPU.
Data analysis	cryoSPARC v3.3, MotionCor2.1, Phenix 1.20, Coot 0.9.6, Pymol 2.0, ISOLDE-1.2, UCSF ChimeraX-1.2, UCSF Chimera-1.14, GraphPad Prism 8.0

For manuscripts utilizing custom algorithms or software that are central to the research but not yet described in published literature, software must be made available to editors and reviewers. We strongly encourage code deposition in a community repository (e.g. GitHub). See the Nature Portfolio guidelines for submitting code & software for further information.

Data

Policy information about availability of data

All manuscripts must include a data availability statement. This statement should provide the following information, where applicable:

- Accession codes, unique identifiers, or web links for publicly available datasets
- A description of any restrictions on data availability
- For clinical datasets or third party data, please ensure that the statement adheres to our policy

All data produced or analyzed in this study are included in the main text or the supplementary materials. A reporting summary for this article is available as Supplementary information file. Density maps and structure coordinates have been deposited in the Electron Microscopy Data Bank (EMDB) and the Protein Data Bank (PDB) with accession codes EMD-35913 and 8J18 for 3-OH-C12-GPR84-Gαi complex. Source data are provided with this paper; EMD-35914 and 8J19 for LY237-GPR84-Gαi complex; and EMD-35915 and 8J1A for GPR84-Gαi complex with no ligand modelled.

Human research participants

Policy information about studies involving human research participants and Sex and Gender in Research.

Reporting on sex and gender	N/A
Population characteristics	N/A
Recruitment	N/A
Ethics oversight	N/A

Note that full information on the approval of the study protocol must also be provided in the manuscript.

Field-specific reporting

Please select the one below that is the best fit for your research. If you are not sure, read the appropriate sections before making your selection.

☒ Life sciences ☐ Behavioural & social sciences ☐ Ecological, evolutionary & environmental sciences

For a reference copy of the document with all sections, see nature.com/documents/nr-reporting-summary-flat.pdf

Life sciences study design

All studies must disclose on these points even when the disclosure is negative.

Sample size	Sample sizes were not predetermined by statistical methods. Sample sizes of cryo-EM data were determined by availability of microscopetime. For functional assays, three biologically independent experiments (n=3) were performed as depicted in related Figure legends. Data were analysed by fitting variou ligand concentrations and readouts using appropriate equations in GraphPad Prism 8.0.
Data exclusions	No data were excluded
Replication	Each experiment was reproduced at least three times independently. Experimental findings were reliably reproduced.
Randomization	No Randomization was attempted or needed. Randomization was not necessary as the independent variables to be tested were sufficient for functional interpretation within this study
Blinding	Blinding is not necessary or valid for the purposes of structural determination.For cyro-EM study, purified receptor-G protein complex samples were applied onto a glow-discharged holey carbon grid and subsequently vitrfied using a Vitrobot Mark V.Cryo-EM imaging wasperformed on a Titan Krios equipped with a Gatan Summit direct electron detector.The microscope was operated at 300 kV acceleratingvoltage. For functional analysis, blinding was not necessary due to the quantitative nature of the experiment . All xperimental data acquired oranalyzed in this study are included in this published article, and subjected to statistical analysis whenever necessary.

Reporting for specific materials, systems and methods

We require information from authors about some types of materials, experimental systems and methods used in many studies. Here, indicate whether each material, system or method listed is relevant to your study. If you are not sure if a list item applies to your research, read the appropriate section before selecting a response.

Materials & experimental systems

n/a	Involved in the study	
☐	☒	Antibodies
☐	☒	Eukaryotic cell lines
☒	☐	Palaeontology and archaeology
☒	☐	Animals and other organisms
☒	☐	Clinical data
☒	☐	Dual use research of concern

Methods

n/a	Involved in the study	
☒	☐	ChIP-seq
☐	☒	Flow cytometry
☒	☐	MRI-based neuroimaging

Antibodies

Antibodies used	scFv16 was used for complex stabilization. The sequence of scFv16 was reported by Maeda et,al. DO1:10.1038/s41467-018-06002-

Antibodies used	w.The gene was cloned into pFastBac vector (Thermo Fisher) and co-expressed with receptors-Gi protein complexes in Spodopterafrugiperda sf9 insect cells (Invitrogen) using the baculovirus method (Expression Systems).
Validation	scFv16 came from an antibody (mAb-16) from Mus musculus, that can binds and stabilizes the Gi heterotrimer. This antibody was generated by Roger Dawson at F Hoffmann-La Roche Ltd (Roche). The characterization of scv16 was reported by Maeda et, al. DOI:10.1038/s41467-018-06002-w

Eukaryotic cell lines

Policy information about cell lines and Sex and Gender in Research

Cell line source(s)	sf9 cells were obtained from Expression Systems (Cat 94-001S) and HEK293 cells were purchased from the American Type Culture Collection (ATCC, CRL-1573).
Authentication	None of the cell lines have been authenticated.
Mycoplasma contamination	All cell lines were tested negative for mycoplasma constamination.
Commonly misidentified lines (See ICLAC register)	No commonly misidentified lines were used.

Flow Cytometry

Plots

Confirm that:

☐ The axis labels state the marker and fluorochrome used (e.g. CD4-FITC).

☐ The axis scales are clearly visible. Include numbers along axes only for bottom left plot of group (a 'group' is an analysis of identical markers).

☐ All plots are contour plots with outliers or pseudocolor plots.

☐ A numerical value for number of cells or percentage (with statistics) is provided.

Methodology

Sample preparation	Sample preparation listed in Methods.
Instrument	Guava easyCyte 8HT (Merck Millipore)
Software	Guava software 2.1
Cell population abundance	Approximately 10,000 cellular events were collected and the total fluorescence intensity of positive expressior
Gating strategy	Gating was determined by the fluorescent intensity of FITC to differentiate positive cells and all other cells.

☒ Tick this box to confirm that a figure exemplifying the gating strategy is provided in the Supplementary Information.
